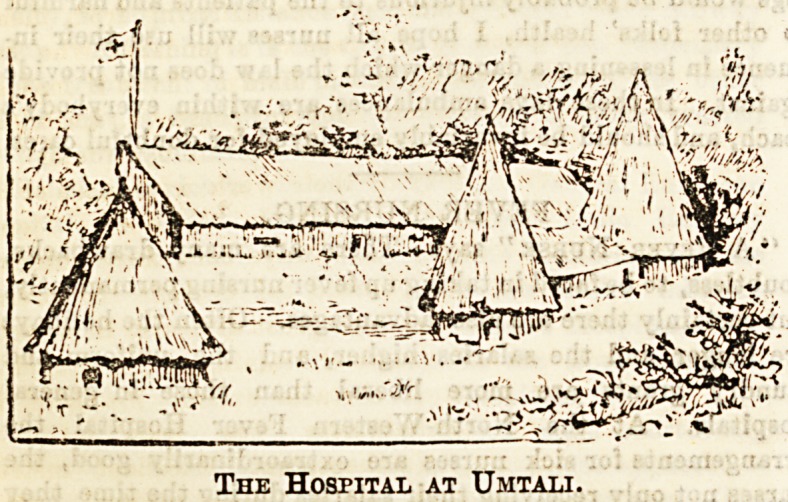# The Hospital Nursing Supplement

**Published:** 1893-05-27

**Authors:** 


					The Hospital, May 27, 1893. Extra. Supplement.
ISfoStHtal" Hurstng itttvvov*
Being the extra JNubsing Supplement of "the hospital ' i-sEwafAfun.
[Contributions (or this Supplement should be addressed to the Editor, The Hospital, 428, Strand, London, W.O., and should have the word
?? Nursing'* plainly written in left-hand top corner of the envelope.]
IRcws from the murstng Twiorio.
THE NURSE DOLLS.
The recent notices in The Hospital relative to the
little Nurse Dolls, appear to have been perused by a
vast number of readers in all parts of this and other
countries. Many pleasant letters have reached us
promising additions to The Hospital Collection of
nursing uniforms. Abundant evidence that the interest
is not confined to one sex is given by certain doctors
having defrayed the cost of some of the dolls which
have been dressed by nurses. Moreover, the costumes
are not only good as "representative uniforms/' but
thev will form an exhibition of exquisite needlework.
Fingers capable of fashioning such beautiful miniature
garments may well belong to proficients in the art of
skilled nursing. Dolls should be sent before June 17th to
Editor, The Hospital, 428, Strand, marked " Dolls/'
in left hand bottom corner of address card.
FALSE ECONOMY.
"While the doctor is examining the patient, the
patient is examining the doctor," writes an American
physician. With equal truth we may assert that the
Bame thing occurs with regard to the nurse. On
entering a ward the silent man and nervous woman
anxiously take stock of the skilled attendant on whom
so much of their future comfort depends. A word of
sympathy, a kindly greeting, may lighten many a
weary load. It is a pity that busy people incline to
economise their courteous acts and words, and it is
short-sighted policy to be saving of small kindnesses.
Lavish expenditure in this, is always repaid a hundred-
fold by gaining the confidence of patients and securing
influence over their somewhat injudicious friends. The
brusque manners of many good women are apt to bring
discredit on even virtue itself.
THE WORTH OF A FINGER.
It is difficult to understand why the loss of one finger
should incapacitate a nurse for following her pro-
fession. Perhaps a surgical nurse might find difficulties
in such matters as bandaging, &c., but a small-pox or
fever nurse ought to be a very useful woman if she has
but nine fingers left for working with. One of the best
accident nurses we ever knew was short of two fingers,
and she was so clever in the use of her remaining eight
that many people never found out the deficiency in
numbers. An accident with machinery not infre-
quently deprives a mechanic of one or more of his
useful fingers, and yet he contrives to follow his calling
?with wonderful skill. In the face of such facts, we are
puzzled by the Small-pox Hospitals Committee con-
sidering that a woman who has unfortunately lost one
finger is therefore prevented from performing the
duties of a nurse. No one will question the kindly
action of the committee in granting a year's salary
(?32) as compensation for the accident; it seems hardly
necessary, however, to decide that the loss sustained
unfits a woman for acting as a small-pox nurse.
DOES MASSAGE PAY?
When people with no knowledge of the subject begin
to talk about massage, curious statements get into
print. One authority decides that " the thing is over-
done," and another asserts that the work is remunera-
tive and easily learnt by women of small education and
no nursing experience. Certainly, a masseuse receives
a fair rate of payment for her work, but, like all other
employment, it is not always easy to get. Many doctors
prefer engaging women who are trained in nursing as
well as massage. Most hospitals and institutions have an
adequate number of duly certificated and experienced
masseuses to supply all the demands of their medical
staff. Therefore, anyone who thinks that a few
inexpensive lessons in the art will secure for her a
certain income, had better, ascertain beforehand how
many medical men she can count upon to send her
cases. Much practice and natural aptitude, as well as
a long and complete course of instruction, are in every
case needed to evolve a competent masseuse.
THE PLAGUE OF FLIES.
Flies are seldom welcome visitors, and they are-
always irritating to sick people, who specially attract
their attentions. Many devices are resorted to, and
the watchful nurse imposes barriers of various sorts
between the intruders and their victims. But our
inherent antipathy to flies is certainly justified by the
results of experiments recently made by Dr.
Sawtechenko, who gives it as his opinion " that flies
may act not only as carriers of the morbific agent of
cholera, but also as hosts of the bacilli." And " the
moral of that is," that we must aim at the extermina-
tion of the little pests! Instead of contenting our-
selves with keeping them away from our sick folks we
should make it our business to get entirely rid, as far as-
possible, of this and every other source of possible
evil.
SAVE ME FROM MY FRIENDS!
This will soon be the petition of the trained nurse of
to-day. She feels herself rapidly becoming ridiculous
by means of her champions. Oddly enough, the latter
generally seem to be absolutely ignorant of the elements
of nursing, and therefore, not only do they discover
imaginary, but they also elaborate and invent
grievances. In the face of such advocates the estab-
lished nurse may well wonder at her own identity, and
question whether she exists for tne patients' benefit, or
if the patients are merely tolerated for her sake. It is
becoming far too common to judge hospitals and other
institutions from the nurses' standpoint, although a
little reflection shows that " Patients and their treat-
ment" may well take precedence of " Probationers and
their privileges." If the two things were more
adequately balanced we should find that many BO-called
grievances were non-existent.
lxxxii THE HOSPITAL NURSING SUPPLEMENT. May 27, 1893.
LADIES' SANITARY ASSOCIATION.
Valuable as are the facts set before us in the many
reports which each week brings to our notice, very few
of the pamphlets can be classed as interesting litera-
ture. The Ladies' Sanitary Association, however, is an
exception, for its thirty-fifth annual report gives an
excellent record of work well done and[so pleasantly set
before us that our interest is sustained from the first
word to the last, and we find ourselves warmly sympa-
thising in this most useful branch of women's work.
All mothers and girls who desire to know more of
this old-established association, with which the name
of Charles Kingeley will ever be closely connected,
should go and see the lady secretary, at 22, Berners
Street, W.
TRAINS AND TRAINED NURSES.
"Whilst the fashion of other garments changes
frequently, and one startling novelty replaces another,
nurses' uniforms are subject to but few modifications.
They are so distinctive in character that the best of
them do not admit of improvement. They are
" suitable," and perhaps that simple word expresses
the highest praise winch can be paid to any dress,
whatever its other merits. * To be suited to the wearer,
a costume must also be convenient for the occupations
and recreations of the woman. It hardly seems as if any
intelligent being could say that a trained skirt was in
keeping with the profession of nursing. Yet whatever
else may change this abuse continues. What a small
proportion of the uniformed figures daily encountered in
the streets, wear gowns which clear the pavements ! Of
late London has been comparatively clean, though the
streets are never so pure as to incline fastidious women
to personally polish them. Again and again attention
is attracted by the nurse, neat in figure and scrupulous
in care of face and hair, collar and hands, yet so utterly
indifferent to antiseptic principles that her dress of
cotton, zephyr, gingham, serge, or merino is collect-
ing as she walks a number of " unconsidered trifles "
from causeway and carriage road. Of course there is
nothing to prevent other women from contaminating
their own skirts, shoes, and stockings with the im-
purities of the streets, but nurses at least are not
free agents; they are under a moral obligation to
isolate themselves from all avoidable sources of in-
fection, and to promote a high standard of hygiene and
sanitation by example and precept. The latter is
surely valueless without the former, and how can any
woman conscientiously assert that she has done her
best for patients to whom she has wilfully imported a
varied assortment of germs ? The much-vaunted
modern " training" is still an imperfect system in
every institution where sweeping skirts are permitted
to its nurses.
A GOOD EXAMPLE.
A women's association, to be managed by a com-
mittee of working women, has recently been organised
at Abinger for the purpose of collecting funds for the
Guildford Hospital. Many persons in this district
annually benefit by this hospital. About sixty women
have cordially joined the present scheme. A house
to house visitation, carefully and tactfully carried out
by Miss Baker, who has been working as district nurse
in Abinger during the winter, originated the associa-
tion. We cordially sympatliise with a movement which
we hope to see generally followed.
NURSING AT HINCKLEY.
It is, indeed, pleaBant when a Committee is able to
say in its second annual report that " both hospital and
district nursing have passed from an experiment into a
well-tried certainty." This is the gratifying conclusion
arrived at by the organisers of the Hinckley Cottage
Hospital and Nursing Institute, and they are certainly to
be congratulated on their success. Sixty-four patients
have been treated in the hospital during the last year,
some of them very serious cases. The report of the
4,554 visits paid by the district nurse during the year
show how much her services are demanded and appre-
ciated.
FROM CHURCH TO WARD.
One of the gifts for sick people, which custom and
common sense alike acquiesce in, is the " new-laid egg."
Realising this, the Rector of Ashford has instituted an
annual offertory on a Sunday in spring, when all the
members of the congregation are invited to bring fresh
eggs, and on the last occasion 610 were collected and
transferred from the church to a London hospital. This
is surely a most excellent and practical form of charity,
and one which might well be largely followed.
DISTRICT NURSING AT YARMOUTH.
Two trained nurses are employed at Great Yarmouth
by the Christmas Charity Trust Fund, and their ser-
vices are in great request amongst the Bick poor. The
third annual report tells of 211 cases nursed, and
4,798 visits paid, and also records welcome gifts of
nourishing food and loans of nursing appliances
through the instrumentality of the ladies' committee.
RIO JANEIRO.
An Anglo-American Hospital has been opened this
year at Rio Janeiro, containing thirty-five beds. It has
an English Matron and two nurses, and a separate
block of building provides space for a steam disin-
fector and a laundry. Great pains have been taken to
render the system of drainage perfectly satisfactory,
and the institution promises to be an immense boon to
the patients for whom it has been erected.
SHORT ITEMS.
The Kent and Canterbury Institute wisely en-
courages the nurses in its employ to join the Royal
National Pension Fund by granting aid towards their
premiums.?A piano has been purchased Jfor the nurses'
recreation room at the Royal Berks Hospital with
donations received from those who have availed
themselves of the services of the private staff. The
Hartlepool Nursing Guild, in issuing its third annual
report, suggests that if the employes at all the works in
the town would contribute a small sum weekly it would
become possible to secure the services of a second dis-
trict nurse.?Lady White has accepted the office of
President of Lady Roberts' Nursing Fund, and Colonel
John Robertson, of Simla, is the new Honorary
Secretary.
"THE HOSPITAL" ENDOWED BED.
This week we have the pleasure of announcing a
handsome donation of ?5 for the Nurses' Bed from
Miss Blennerhassett (Sister Aimee). She has written a
most cordial letter from Umtali in which she says,
" The Nurses' Bed is a splendid idea, and I hope you
will get a large number of subscribers." A sympathetic
communication from a nurse living abroad is always wel-
come, and her example will be, doubtless, approved and
followed by other workers who have an equally keen
interest in sick or weary fellow nurses. The nurses
at Fitzroy House, have sent ?2, making the total
amount received up to date is ?26 9s., and ?3 lis. is,
therefore, still needed to maintain the free bed for
another year. All sums sent to the Editor, 428,
Strand, will be immediately acknowledged.
May 27,1893. THE HOSPITAL NURSING SUPPLEMENT. lxxxiii
Sanitation for Burses.
By a Sanitary Inspector.
VII.?NOTIFICATION IN FOREIGN COUNTRIES.?
FINLAND.
In Finland we find, as regards sanitary matters, a curious
mixture of enlightenment and ignorance. Owing to its
proximity to Sweden, and to the fact that in early days the
two countries were one, the two formerly advanced pari
passu. Now, however, Finland has been -left far behind
Sweden, and will have to introduce many and great improve-
ments as regards sanitary matters before it comes up to the
standard of most other European countries. The climate is
said to be extremely healthy, and hence there is no reason
why epidemics on a largo scale should decimate the inhabi-
tants of that favoured land, nor why typhoid fever, for
instance, should be bo prevalent, and the mortality from] it
bo large. But because Nature has done so much for the
country it hardly seems a valid excuse for man not to exert
himself to the utmost. On the contrary, there should be
every incentive to do all that can be done to make Finland
as nearly a paradise on earth as possible, and to stamp out all
diseases which arise from defective sanitation. Although
something has been done of late years, and the Government
and the people have awakened somewhat to the importance
of sanitation, there still remains much to be completed. In
1879 a new code of hygiene was framed, and more recently still
the University in the capital, Helsingfors, has recognised the
importance of this science, especially for medical students,
and has established a Professorship, and a regular course of
study in it.
At present the chief fault that has to be found is that this
country does not fully realise the great necessity for taking
every possible precaution against the spread of infectious
diseases. The first step should be for the Government to
awaken to this important faot, and then to set to work and
draw up a Public Health Act or Code of regulations, with
special reference to infectious diseases, epidemics, notifica-
tion, and disinfection. After the laws have been framed,
then duly qualified persons must be appointed to see that
these regulations are properly and efficiently carried out.
The result of suchaction would doubtless be to further reduce
the present high rate of mortality, which has already fallen
somewhat since the introduction of the improved Hygiene
Code of 1879, but;is still far too high, both as compared with
that of other nations and especially when the favourable
natural conditions of the country ,are taken into account.
The supreme authority in hygienic matters belongs to the
chief of the Civil Department in the Senate, and under him
is the Medical Council, which undertakes the immediate
direction and control. It is composed of a president and
three members, besideB numerous assiatanta and others, who
carry out the duties of special branches. A unique
characteristic of this Council is that it haB also five district
?doctors established in1 Helsingfors, of whom one is actually a
qualified woman. The duty of these is to care for the aick
Poor, under the immediate direction of the commission.
Instead of having several inspectors,"each with a special dis-
trict, and frequent inspections and reports made, the direotor
of the Medical Council has liimBelf to go on a round of inspec-
tion throughout the country, and has so to arrange his visits
that the whole is inspected onoe every two years only. An
arrangement which muBt evidently admit of many abuses
occurring in the intervals. The other dutieB of the Council
comprise the control of hospitals, asylums, orphanages, dis-
pensaries, druggist shops, vaccination stations, and bathing
CatabliBhmentB. For each province there is a provincial
doctor. His duties seem to have been laid down with great
minuteness in 1832, and not to have been'materially altered
since then. In case of an epidemic of infectious diseas
breaking out, the provincial doctor has to make the neces-
sary regulations to prevent its spread, and to make arrange-
ments for nursing patients at their own homes. He has the
control of midwives, apothecaries' shops, and vaccination
stations, and is expected to look after any mineral springs
existing in his district?and all these he must systematically
inspect once a year. The provincial doctor is both appointed
and paid by the Government.
Each town has a Health Commission, composed of the chief
municipal doctor, the chief of police, an engineer, with
a president and vice-president. This Commission has charge
of all the sanitary regulations of the town, and is required to
inspect all factories, workshops, workmen's dwellings,
restaurants, furnished rooms, and even private houses under
certain conditions. In rural communes or cantons, the
sanitary authority is the Communal Council, which has to
furnish a detailed annual report of the state of health in the
commune, and of all improvements which may have been
made. The powers of this Council resemble those of the
Municipal Health Commission, only, of course, on a smaller
scale, and confined merely to the commune. In Finland,
notification is not made compulsory by law, nor is there any
list of diseases which should be notified. However, in the
event of an infectious disease breaking out in a private house,
the father of the family, or failing him, the owner of the
house, is expected to report the same without delay to the
Health Commission, whose duty it is to take the necessary
precautions agaiuBt the spread of infection.
In the time of epidemics the power that the Health Commis-
sion can exercise is greatly increased and extended. It can
divide up the district into small divisions, and place an in-
spector over each ; it may appoint more doctors if the present
supply is not deemed sufficient. Ic is also required to distribute
amongst the people simple pamphlets to teach them how to
avoid infection, and how to treat the patient till the doctor
can arrive, and other useful knowledge of a similar kind. In
the cantons the president of the Communal Council must give
information to the provincial doctor, who is responsible for
the necessary measures to prevent the spread of the infectious
disease.
There are no special hospitals for infectious diseases in
Finland, but the new hospital in Helsingfors, built in 1888,
has a pavilion set apart for isolating infectious cases. How-
ever, as it only contains fourteen beds, and the population is
as nearly as possible 60,000, it is evident that the accommo-
dation is very deficient, and it is also clear that all the
arrangements for the prevention ef infectious disease are
consequently most incomplete and unsatisfactory, and as re-
gards country districts there appear to be no measures at all
taken to isolate infeotious disease. There is no regular and
well-considered system of disinfection in fever, nor is dis-
infection compulsory, and hence is more often neglected
than not. There are no measures in force for checking the
spread of infectious diseases in Bchool, and consequently these
mostly take an epidemic form.
The regulations as laid down by the various sanitary
authorities are good, but remain for the most part in-
effectual. because those who have to carry them out do bo
inefficiently, if at all, either because they are indifferent or
ignorant. The only remedy is that notification and disin-
fection shall be made compulsory by law, and fines imposed
for their neglect.
Then we Bhould not have such dreary records as those
furnished by Dr. Palmberg's tables of mortality from typhoid
fever in Finland. By these we find that whereas in England
and Wales the mortality from typhoid fever was, in 1890,
1*9 per 10,000 inhabitants, in Finland it was for the same
year as high as 2*54 per 10,000 inhabitants.
lxxxiv 7HE HOSPITAL NURSING SUPPLEMENT. May 27, 1893.
TRurslng in 3nbta.
THE DUFFERIN HOSPITAL, ALLAHABAD.
By Our Indian Correspondent.
This hospital is a very pretty red-brick building standing
in its own enclosure and looking very much like a cottage
hospital in England. It lies between the Kuslim Bagh and
the city, at no great distance from the entrance to the railway
station. Adjoining it Ib the Colvin Hospital and Dispensary
for the treatment of male patients, and again on the other
side of that, a beautiful new eye hospital is being [erected
through the generosity of Monolim Das.
Allahabad is rich in hospitals; there is a General European
Hospital, a Police Hospital, the Ease India Railway
Hospital, the present Eye Hospital, the Dufferin, and the
Colvin. In addition to these there are under military
government, the Station Hospital, Women's Hospital, Can-
tonment Hospital, Followers'Hospital, the Fort Hospital,
Native Infantry and Native Cavalry Hospitals, &c.
On visiting the Dnfferin Hospital we were courteously
received by the lady doctor who is,in charge; she appeared to
be house surgeon and lady superintendent in one. The
nurBes are all either natives or Eurasians. The Purdah wo-
men, who belong to the high Hindu castes, have separate
accommodation from the other patients; they have each a
cottage, one of a row, behind a high wall; the male doctors
never see or visit them. There were only two Purdah
patients in, and we saw one of them. Her little room was
plainly furnished and she lay on a charpoy, her children were
playing on the ground, and her mother-in-law was in atten-
dance preparing her food. The patient was consumptive,
sadly young looking to be the mother of a family. We next
visited the private wards. In each was a lying-in case. The
rooms were exactly like private wards in England. In one
was an Eurasian woman who showed us with great pride her
little black baby. In another, a strolling actress with her
young baby ; she was a white woman, a pretty American, and
she looked languid and as if she was making but a slow re-
covery. A few days afterwards a flaming fly-bill announced
her appearance a fortnight hence, in " her famous trapeze per-
formance !"
We then went into the two general wards. One was called
the European ward, but the inmates happened to be vary
black, owing to the overflow of the native ward. They had
beautiful btds and spring cots. The other cases consisted of
tubercular disease, pelvic abscess, hepatic abscess, &c.
The dispensary, presided over by a native female com-
pounder, was a picture of cleanliness and order. It was
well arranged and well stocked, It would have gladdened the
eyes of many dispensers in Eaglish hospitals.
The operation theatre, which was entered from a spacious
waiting-room, with its acreened-off corners (part of it
furnishing an office), was a model of light and ventilation,
and would accommodate a large number of students. There
was an excellent cupboard with glass doors, to hold the
instruments, the obstetric instruments holding a prominent
place. They had one or two large tables, which are a great
convenience when there is space for them. The operation
table was covered with spotless mackintoshes.
The lady doctor told us she had performed a number of
operations during the year herself. Except for the patients
being so dark and the nurses uncommonly black, there was
nothing particularly different from English hospitals.
Certainly the Dufferin hospitals are doiDg grand work, and
are well worthy of support This small hospital of about
thirty beds, during the year 1892 received 452 indoor and
12,428 outdoor patients, making a daily average of over 26
in-patients and over 91 out-patients. The staff consisted of
a lady doctor, Miss Dissent; a midwife, Mrs. Phulmain
Das; a matron, a dresser, two nurses, and servants.
Although the hospital is not in debt, funds are needed to
build quarters for the matron, nurse*, and destitute police
caies.
XTbe leper Hsplum, Colombo,
Ceylon.
By a Colombo Resident.
This asylum, one of the oldest in the East, was founded by
a Dutch lady, herself a leper, who left her property as a free
gift to Government, at the time of the British occupation of
the Colony, for the purpose of founding a home for lepers.
It ocoupies a beautiful site at Hendala, four miles from the
fort,[at the mouth [of the Kelan6 river, 'within view of the
sea and the entrance to the harbour of Colombo. It has,
accordingly, all the hygienic advantages combined with the
necessary conditions for segregation which its situation and
environments afford for a leper hospital.
The grounds, about 17 acres in extent, have a fine river
frontage, the Hamilton Canal forming the western boundary.
They are well laid out, with palms, shrubs, and large trees,
neatly trimmed paths, and shaded lawns, presenting much
the appearance of a park in England. The buildings are
detached and on the pavilion system, consisting of fourteen
wards,^with accommodation for 228 patients, and an infir-
mary with twenty beds for the separate treatment of Bick
lepers.
Most of the wards are recent additions to the asylum,
and are well lighted and ventilated, with ample breathing
space and accommodation. Attached to them by corridors
are the necessary ablution rooms, with hot and cold baths,
and closets on the dry earth system. The female wards are
separated and isolated from the male by a partition wall.
The administrative buildings comprise the medical officer's
quarters, office and dispensary, dispenser's and steward's
quartera, nurses quarters in the female enclosure, mortuary
and post mortem room, kitchen, provision stores, and stores
for bedding and linen.
There is in addition a fino little chapel prettily situated on
the riverside, the gift of Mrs. Copleston, the wife of the
present Bishop of Colombo, for Church as well as
denominational services. A Roman Catholic chapel, dedi-
cated to St. Xavier; and a pansala and wihare for the
Buddhist inmates of the asylum. A reading room and a
recreation room, with a school attached for instruction of the
leper boys, complete the structural details of the asylum.
The dieting is on a liberal scale, admitting of great variety
to Buit the different classes of inmates.
An amply supply of good potable water is obtained from,
the town mains by connecting pipes laid across the river,,
and from the river itself for general purposes of ablution.
The sewerage is daily incinerated.
The staff of the asylum consists of a resident medical
officer, a steward, dispenser, and fourteen ward attendants,
with an Eurasian matron who has supervision of the female
wards.
Exceptingthe matron, there are no nurses attached to the
hospital, the nursing being done by the ward attendants,
who are untrained to the work.
One hundred and eighty-eight male, and forty.two female
lepers are at present under treatment.
Of the recognised types of the disease, nerve leprosy'
appears in a larger proportion of cases than the tuberculated
and mixed varieties, which are more prevalent in cold
countries.
As regards the duration of the disease, there are several'-
patients who have been resident in the asylum for more than
thirty years. The oldest patient, a case of nerve leprosy, is
aged 80, the youngest two years. Admission is not compul-
sory, nor is any restriction imposed as to the discharge of
patients, but every elfort is made by kind treatment, good
food and shelter, to retain them in voluntary segregation in
the asylum.
May 27, 1893. THE HOSPITAL NURSING SUPPLEMENT. lxxxv
As regards |the growth and extension of the institution,
the records show during the decennial period 1802-11 only
three lepers were under treatment, while during the last
decennial term ended December, 1892, the daily average
in the asylum was 177'54. But the disease, which is endemic
in the island, is making no real progress in proportion to
population, and the number of lepers as far as ascertained at
present, cannot be considered alarming, or calling for im-
mediate legislative interference with a view to compulsory
segregation.
Although every known treatment has been adopted in the
hospital, no absolute cures have been recorded, but a large
number of patients have been benefited and resumed their
ordinary avocations, and a great many more are now in the
asylum in fairly good health and condition, without the
appearance of fresh symptoms or any farther progress of the
disease for some years.
It may be remarked in conclusion that the asylum is
entirely supported by Government at an annual cost to the
colony of ?35,000, and that the successful working of the in-
stitution is largely due to the unwearied efforts of the senior
medical officer, Dr. Myer, who for many years has devoted
his time and talents to the cause of the lepers. The chap-
lain, Mr. Arndt, is equally well-known as a true friend to
the unfortunate sufferers, whose long residence in the hos-
pital is compelled, not by law, but by the irresistible
attraction of the kindly spirit which reignB within the walls.
lonbon Ibospital.
STUDENTS' CONCERT.
The beautiful Library at the Medical College was filled with
a large and enthusiastic audience on the occasion of the ex-
cellent concert last week. The hall was tastefully decorated
with palms, flowering plants, &c., and amongst the audience
were members of the Medical Staff and the Hospital Com-
mittee, as well as many students and nurses with their
friends. The programme was a very good one, and the
concert committee, of which Dr. Gilbart Smith is chairman,
must certainly be congratulated on the admirable arrange-
ments, which secured a delightful evening's entertainment
for their numerous guests.
IHoveltles for IRurses.
BOOTS, SHOES, AND COSY TEAS.
The London Shoe Company has opened some fine premises
at 11G and 117, Bond Street. The large, airy rooms are
elegantly fitted up, and are most conveniently situated on
the ground floor. Shoes of every possible shape and of many
shades of colour are contained in the boxes, which entirely
line the high walls. Not only shoes for ordinary and festival
?ccasicns, but also for ward wear, are offered at equally
moderate prices. The Salon dt Rendezvous is a special
feature of this luxurious establishment, and a liberal supply
?f illustrated papers and journals is arranged for ladies, who
are glad to rest in the intervals of a day's shopping. Light
refreshments are provided at low rates, and on the next floor
comfortable and convenient dressing-rooms are prettily fur-
nished. Numbers of visitors to town will gladly avail them-
selves of the accommodation offered, and it will probably suit
many nurses to know of a pretty, quiet WeBt-end room where
friends can enjoy the luxury of tea and talk amongst har-
monious surroundings. The club rules are very simple, and
tickets are only iEsued to ladies who have offered "unim-
peachable references." The number of members is to be
limited, and after the limit is reached, application is to be
taken in rotation.
Ewerpbobp's ?pinion.
[Correspondence on all sub'ects is invited, but we cannot in any way
be responsible for the opinions expressed by our correspondents. No
communications can be entertained if the name and address of the.
correspondent is not given, or unless one side of the paper only be
written onj  ??
MEASLES.
"D. P. H." writes : Measles not being scheduled in Eng-
land, persona suffering from the disease can be conveyed in.
cabs without any risk of fine. As, however, such proceed-
ings would be probably injurious to the patients and harmful
to other folks' health, I hope oil nurses will use their in-
fluence in lessoning a danger which the law does not provide
against. In these days ambulances are within everybody's
reach, and should be invariably employed for doubtful cases.
FEVER NURSING.
" A Fever Nurse " says: There are many drawbacks,,
doubtless, to be faced in taking up fever nursing permanently,,
but certainly there are also advantages. Often the holidays
are longer and the salaries higher, and the uniform and
laundry grants are more liberal than those in general-
hospitals. At the North-Western Fever Hospital the
arrangements for Bick nurses are extraordinarily good, the
nurses not only receiving their salaries during the time they
are laid up with illness contracted from patients, but also
gettiDg an allowance of 30a. per week extra to enable them to
go into the country to regain their strength. This was men-
tioned publicly by Dr. Gaton, the medical superintendent,,
the other day, and should certainly be noted by all nurses
who have suffered from a very different line of treatment in,
other institutions.
TKIl here to (Bo.
An Illustrated Recital with musical accompaniment of
D. G. Rosetti'a " The King's Tragedy " will take place at.
Queen's Gate Hall, on June 1st and 2nd, at 9 p.m. Tickets
to be obtained from Miss Gertrude Stewart, 29, Parliament.
Street, and the usual agents.
A matinee is to be held at the Trafalgar Square Theatre
on May 31st in aid of the Poplar Hospital for Accidents.
The performance promises to be an attractive one. At a
previous matinee in aid of the hospital at the same theatre
Messrs. Penley and Chevalier gave their services. The
performance was well patronised, and proved a great success,.
?500 being added to the funds of the hospital thereby.
A matinee In aid of the Charing Cross Hospital is also to-
take place on the Bame day, May 31st, at the Shaftesbury
Theatre.
A grand evening concert is to be held at St. James's Hall
on June 6 th in aid of the Dental Hospital of London. Many
well-known artistes have promised to assist.
The Graphic Society of St. George's Hospital open their
interesting annual exhibition on May 30th. Past and pre-
sent members of the Medical College are well represented,
amongst the exhibitors; lantern demonstrations are to
be held on Wednesday, May 31st, and Thursday, June 1st,
at half-past eight. The Hon. Secretary, Mr. Chas. Slater*
will give any information required.
Hppotntments.
[It is requested that successful candidates will send a copy of their
Applications and testimonials, with date of election, to Thb Edizob,
The Lodge, Porchester Square, W.]
Kenilworth Convalescent Home.?Miss Kathleen B..
Beamish has been appointed Matron of the Kenilworth'
Convalescent Home. Miss Beamish was trained at the
Borough Hospital, Birkenhead, and was for^ eight years
Sistar of the Female Medical Wards at Bristol General
Infirmary. We wish her success in her new work.
Ixxxvi THE HOSPITAL NURSING SUPPLEMENT, May 27, 1893
IRurslng in tbc Xanb of ?pbir.
THE HOSPITAL AT UMTALI.
The little sketch given herewith certainly depicts a very
different scene from any of those usually brought to our
minds by the word " hospital." Yet this is the picturesque
erection which bears that designation in Manicaland, and In
it a great deal of good work has been done by Sister Aim6e
and her companions. In the laBt Christmas number of The
Hospital we published an interesting description of a
nurse's life at Umtali, accompanied by an amusing Bketcb.
Our readers will certainly welcome the present picture of
the head quarters of the nurse whose liberal donation to " The
H ssp ital End owed Bed " we report in the usual column.
ttbe 1Ro\>aI British IRurses'
association.
At a special meeting of the council of this As-
sociation, held on the 24th inst., Sir William Savory,
Bart, F.R.S., in the chair, Sir Joseph Fayrer,
Sir Dyce Duckworth, Dr. Bezley Thorne, Dr. Gaza
Brown, and a goodly number of the nurses being present,
H.R.H. the Princess Christian announced that a Royal
Charter would be granted to the Association by the Queen in
Council. The Princess expressed great gratification that Her
Royal Highness should be able to make this statement on the
Queen's birthday, hoped that the work of the Associa-
tion would tend in many ways to promote the welfare
of the nurses and the good of the public, and expressed the
belief that a register of nurses would prove of great advan-
tage in many ways. Princess Christian Btated, despite the
resolution passed at Edinburgh, that the Council had deter-
mined to adhere to the rule which prescribes three years'
training as the minimum. Sir W. Savory and other speakers
congratulated H.R.H. upon the success which had crowned
her herculean efforts on the part of the British Nurses' Asso-
ciation, and the Princess was much and deservedly cheered
for her devotion to the cause upon which she had set her
heart. No details of the Charter were, however, forth-
coming at the meeting, and there was no discussion.
The following scheme of the Executive Committee for the
Constitution of National Branches of the Association in
Scotland and Ireland was put to the meeting by Sir W illiam
Savory, and adopted unanimously :?
The General Council authorises the formation of branches
of the Association in Scotland and Ireland, with head-
quarters in Edinburgh and Dublin respectively, under some
such regulations as the following : ?
1. The President of the Association shall be president q?
each branch ; the Vice-Presidents of the Association, resi-
dent for the time being in Scotland or Ireland, shall be Vice-
Presidents of their respective branches, and shall, so far aa
circumstances will allow, have the same powers with regard
to their respective branches, as are possessed by the Presi-
dent, with regard to the entire Association.
2. The affairs of each branch shall be managed by a com-
mittee (consisting of an equal number of medical men and of
hospital matrons and nurses), which shall be elected annually
by the members of the branch. The committee first nomin-
ated shall hold office for three yeara, and thereafter, one-
third of the members shall retire annually by rotation, and
shall be ineligible for re-election until twelve months have
elapsed.
3. The branch committees shall frame suoh regulations
for their guidance, and shall meet at such times and places
as may seem to each committee desirable, provided always
that such regulations and meetings shall not contravene the
letter or the spirit of the ordinances or the bye-laws of the
Association. The members of the branch committees shall
be, cx-officio, entitled to attend, speak, and vote at any meet-
ing of the executive committee and of the General Council of
the Association.
4. The branch committees shall have power to admit
applicants, resident in Scotland or Ireland respectively, to
membership of the Association or to registration, who fulfil
the requirements laid down in the bye-laws and regulations
for [the time being in force, subject to such controlling
authority on the part of the executive committee of the
Association as may be imposed by the terms of any charter
which may hereafter be granted to the Association.
5. Each branch shall be governed in strict accordance with
the bye-laws and legal powers and rights of the Association
for the time being in force.
6. The original application forms of all candidates for regis*
tration or membership Bhall be forwarded to the officeB of the
Association in London for due preservation and enrolment.
7. The disposal of the annual or life subscriptions of mem-
bers of the national branches, and the proportions of such
subscriptions and registration fees which shall be contributed
to the, parent Association, shall be determined by agreement
between the executive committee and the branch com-
mittees, when constituted, subject to the approval of the
General Council.
8. Each branch shall hold an annual meeting for the elec-
tion of the branch committee and for such other business
as may be necessary ; and also Buch other meetings as may
from time to time be considered necessary or advisable.
9. The secretary of each branch shall act as local secretary
of the Association, and perform the duties of that office.
10. Each branch shall forward duly audited accounts of
its receipts and expenditure, made up to June 30th in each
year, to the annual meeting of the Association ; and also a
quarterly report of its proceedings to the General Council of
the Association on or before March 31st, June 30th, Sep-
tember 30th, and December 31st in each year.
11. The General Council of the Association may alter,
amend, or add to, any or all of the foregoing regulations as
may from time to time seem advisable.
(Signed) Helena, Chairman.
On the motion of Sir Joseph Fayrer, a cordial vote of
thanks was passed to H.R.H. the Princess Christian, and
the proceedings terminated.
Spain.
Tiie principal hospital in Madrid is a very large one. It is
called the General Provincial Hospital, and was founded in
1587 by Felipe II. The present building was opened in 1781,
and has 42 wards, some of them containing over one hundred
beds, and accommodating altogether 1,500 patients. It is
very easy for a sick person to gain admittance, as the only
formality required is the presentation to the doctor on duty
of the ''Cedula," a kind of passport, which everybody in
Spain is obliged to possess. Therefore the admission of
in-patients is merely after all regulated by the con-
dition of the hospital; which is, unhappily, often overfull-
There are ten -houses in Madrid, one to each district, which
are called Casas de Socorro, and are established for the treat-
ment of all classes of accidents. Advice is also given there
gratis to the working classes between the hours of twelve and
two. The doctors in charge of these houses are empowered
to give orders for the admission of patientB to hospital8
whenever necessary.
?* ' V ''' U' y- M \ ?./??,r? *
Z"*nenJNL\ ? v "? ?/(?',?'' V^vtX* ^"'aiRh
'/: #/. r^-:^ f H5!i 1
\...._* r..u!,
The Hospital at Umtali.
Mat 27, 1893. THE H0SPI7AL NURSING SUPPLEMENT. lxxxvii
for TRea&ing to tbe Stcft.
" WHIMS."
Is it true? Is it necessary? Is it kind? It has been
recommended that everybody Bhoald ask him or herself these
three questions before making'an unkind remark about their
(neighbours, so we will just see if it be true, or necessary, or
kind to Baylthere are such things as " whims " in illness.
First, I think it must be true, because sometimes when one
has sifted the complaints and maladies a self-pitying invalid
has poured into our ears, there seems to be nothing but a
gocd solid layer of "whims," on which the illness is based.
We are not for a moment denying that these hypochondriacs,
these malades imaginaires really suffer what they bewail, but
their fondness of self has aggravated a alight inconvenience
till it has become a great one. They feel a pain somewhere,
and if it is not immediately relieved, they are so sorry
for themselves, that they talk of it and dwell upon it till they
get frightened. Then the mischief fixes itself on the nerves,
and in very bad cases it is only necessary for a bystander, or
?sympathetic friend, to hint at a locality where the pain
anight occur, for the patient to start a violent fit of neuralgia
in the exact spot. This sort of thing ofteneat occurs with
wayward and undisciplined girh, but we also see it in women
-of all ages, women who have never been taught self-control,
who are only grown-up spoiled children'giving way to every
whim that comes into their heads.
I think that what has been said is sufficient to prove
> that it is necessary sometimes to speak of whims in
illness, and to advise those who suffer from them to
get rid of them as fast as possible; while about the kind-
ness of a word of warning there can be no question.
It may hurt the feelings of the person accused of "giviDg
way " to be told there is nothing serious the matter, and
that all Bhe requires is, to use a popular phrase, to '* pull
herself together," but it is cruel only to be kind, as all
medical men agree, that want of self-control produces
hysteria, one of the most difficult complaints to deal with.
The symptoma should be grappled with at the very beginning,
as after a time the poor patient is powerless to help herself.
There are, however, milder forms of whims in which sick
people indulge ; sufferers who are Bufferers indeed will
frequently aggravate their misery by fancies. They fancy
their nurse or attendant is thoughtless of their comfort, or
that their friends are unsympathetic, and are wearying of
their prolonged illness. Then they fidget if the blind is a
trifle too high or too low, and vex themselves because one
friend'8 voice is so loud and strident, it goes through their
heads, while another speaks so low they cannot hear at all,
and so on, &c. Dear friends, do not think us unfeeling, your
real maladies have our deepest sympathy, and we would if
we could relieve you of them entirely. Granted that those
about you are sometimes thoughtless, they are but human.
There is only One who is always considerate, always sym-
pathetic, always close at hand to help us ; try and fix your
love on Him and draw your happiness from His care. It
^ay be that He sees you require these little chafes and
Worries to complete the perfection which His Father by His
chastenings is working in your souls. Do not mar the
plan by grumbling at trifles. Ask the help of your dear
Lord to bear them cheerfully, and they will melt away as the
dew before the rising sun, and your hearts will be filled in-
stead wth that peace which passeth all understanding.
Wants and Mothers.
Qan any reader suggest a home whioh will admit free a gentleman of
mcnrablo and helpless P His father, a clergyman, lias recently died,
Brwing no provision for his affloied son. Particalars can be obtained
om Mr. Wilkinson, ApBley Cottage, Stockport.
H meolectet* ffielb.
MENTAL NURSING.
The general public is now well accaatomed to demanding
trained nurses for medical, surgical, and monthly cases.
For " mental" nursing, that branch which demands special
aptitude and instruction, it still has to put up very often
with only " experienced " women. Experience is a grand
educator but hardly a sufficient one in the case of diseases
of the brain. Why should persons suffering from mental
disorders be relegated to the care of attendants who have
received no special training in the disease? Even if conscien-
tious caretakers of the patient they yet possess no knowledge
whatever of his actual complaint and its symptoms. By
" the light of nature " they do their work with more or less
success, but they actually work in the dark. Mental
nurses, when fully trained, can always earn three guineas a
week, and moreover the demand far exceeds the present
supply. Hitherto there has been great difficulty in securing
complete theoretical and practical training in this branch of
nursing, but now no such obstacle exists. Provision for
the reception of probationers has been made at
Berry Wood Asylum, Northampton. Excellent instruc-
tion, including lectures from the medical staff,
comfortable accommodation, with certificates of proficiency
after adequate training, are offered by the superintendent,
and yet the "new departure" is received without
enthusiasm, nay, worse, with absolute indifference by
hundreds of intelligent young women who complain that
they " cannot get work," in fact, those who make this
lament will not take the painu necessary to fit themselves
for employment. Hundreds of educated girls have to earn their
own livelihood, and many of them grow bitter whiht waiting
for vague " work " which they seem to think ought to fall
into their languid hands. They reproach the world for the
difficulties which beset their paths, and see their small
means gradually dwindling away whilst they grow daily
more desponding. If part of the tiny capital were invested
in learning thoroughly how to do anything well, there would
be a perceptible decrease in the ranks of poor and unemployed
gentlewomen. When a responsible and well-paid occupation
is within reach of all, it 1b discouraging to the promoters of
the Bcheme for recognition of its advantages to be so tardy.
IRotes ant> (Queries.
Queries,
(135) L.O.S.?Will someone tell me the quickest and cheapest way
for an experienced nurse to obtain this diploma??Superintendent.
(186) Mental Nurse.?Pleace give me uddreas of Medico-Psychological
Association. - B.
(137) Dispensing?I shall be much obliged for any information as to
where instruction in dispensing is given to women.?Inquirer.
(1S8) Training.?Oan I get tnoronghly trained in a provincial hospital P
?Probationer.
(139) Lectures.?Do any hospitals permit ontside nurses to attend the
lectures given for the probationers ?? Janet.
(110) Lepers.?A. trained nurse wants information a3 to the best way
of getting work amongst lepers.?Magdalen.
Answers.
(135) L.O.S. {Superintendent).?Write to the Secretary of the L.O.S.,
20, Hanover Square, or consult Matron of any Maternity Hospital.
(136) Mental Nurse (R.)?Inqufries should be addressed to Dr. Spence,
Burntwood Asylum, Lichfield.
(137) Dispensing (Inquirer).?There is a chapter on this subject,
which will tell you all you want to know, in Burdett'fl " Hospital
Annual," 428, Strand.
(138) Training (Probationer).?Certainly you can, when the matron is
herself a thoroughly-trained nurse.
(139) Lectures (Janet).?Yes, it is allowed at some training sohools,
and information on this subjeot oan always be obtained by writing to
t he Matron.
(140) Lepers (Magdalen) .?You must be more explicit in your query.
'Where and when do you want work? Have you private means, or do
you require salary ?
lxxxviii THE HOSPITAL NURSING SUPPLEMENT. May 27 189.1
?be flDuses' Hooklna ?lass.
" Singularly Deluded " * is a brisk and even brilliant tale,
with a suggestion of a psychological problem about it, which
it is part of the scheme of the book to cheat the reader
with. And the reader is cheated, until the last chapter
unravels the mystery, and he finds that it is himself and not
Mr. Leslie Somers who has been " singularly deluded " after
all.
Leslie Somers is a successful barrister, so successful that
when the story begins he has been suffering from over-work,
and has been ordered to take perfect mental rest at the
quiet seaside village of Trewport. Thither he repairs with
his wife, nee Gertrude Wendell, and their three-year-old son.
Before long, the symptoms which caused anxiety have dis-
appeared, and perfect health, physical and mental, seema to
be restored. One day, the little family are walking across a
neighbouring heath, little frequented, although the railway
crosses it, when Leslie finds a rope among the grass, and in
a sportive mood ties his wife to a telegraph posb. He
threatens to leave her there a prisoner, and really goes
away, leaving her laughing at the jest, while the little
boy dances around her helpless form with mischievous
glee. But the jest loses savour when he does not return, and
turns to terror when Gertrude sees a figure which resembles
her husband's?the same height, the same waving red-brown
hair, clad apparently in the same tweed suit, walking
rapidly, and without turning his head, in the direction of
the town. She calle, but he does not hear or does not heed,
and soon disappears from sight. What can she think but
that the over strained brain, which had seemed tojbe recover-
ing, had suddenly broken down?that her husband is mad.
Before long a new terror arises. Her child is playing on
the railway line when she hears a train coming. Vainly
she calls the boy to come to her. " The child, frightened
by her cries, sat up and looked at her, but would not move,
while the long train came on at a terrific rate, rushing
towards him. Shriek upon shriek, shriek upon shriek, the
wretched mother sent up to heaven; and the solid post to
which she was tied rocked with the fury of her struggles,
but the cord did not give an inch. . . . She was conscious,
but she could not stand ; and it was the cruel cord, eating
further into her flesh as her weight sank upon it, which for
the moment supported hEr." The train passes?on the other
line from that where the boy stands, and that agony is
passed. As if it were not enough, however, the child falls
asleep on the line, and another train comes up, this time on
his side of the rails. But he is in the middle of the line, and
it passes above him without touching him. As a matter of
art we think this second train a mistake. The situation
loses power by repetition ; our feelings are^not twice equally
touched by the same incident.
Gertrude is at last released by Lord Wartlebury, an elderly
nobleman, who is yachting in the neighbourhood, and has
stopped at Trewport for the day. He is wandering about
the heath with a friend, Dr. Manseli, when they fiud the
poor lady tied to the post, and conduct her and the child to
her lodgings. Mr. Somers is not there, and inquiries made
by Lord Wartlebury show that a gentleman answering to
his description set out for London that afternoon. Thither
Gertrude follows him, traces him to a hotel, where she finds
he passed under the name of Lawrence Soames, and which
he had left for Waterloo Station, en route for Southampton.
She, too, goes to Southampton, accompanied by an elderly
sister of her husband's, who fully shares her anxiety. They
are sure that this is a case, of a kind rare but not unknown
to science, when through some mysterious blot on the brain
a man forgets his name and identity and seems to start life
* " Singularly Deluded." By Sarah Grand, author of "Ideila," ?' The
Heaveniy Twins," (Edinburgh: Blackwood and Sons. 3 vola.)
anew. The suggestion is made by Dr. Mansell, who with
Lord Wartlebury has accompanied Gertrude to town, and
seems to fit the case to perfection. It is necessary, then, to
keep the patient in sight and overtake hicn if possible, for if
once lost to view Leslie Somers will be no more and Lawrence
Soames may never be discovered.
At Southampton they learn that Mr. Soomea had left for
Guernsey, en route for St. Malo, meaning, he had said, to
have a few days on the Continent before starting for San
Francisco. Miss Somers awaits his return at Southampton,,
while Gertrude, hopiDg to head him, sets out for St. Malo*
But the vessel Bhe sails by takes fire ; forced to choose
between fire and water, she is nearly drowned?is saved,
indeed, only by the Belf-sacrifice of a young man, who givaa
up his hold on the life-belt which is insufficient to support;
the weight of both ; and, picked up at sea, is brought
back to Southampton. A precious day i3 lost ; but nothing
dauntsd she sets out for St. Malo once more. The Guernsey
packet, with Lawrence Soames on board, arrives just before-
her. He has quitted the boat, but he has left his luggage'
to be called for when he should have settled to which hotei
he should go. For five weary hours Gertrude stands on the
quay watching that luggage?meanwhile forgetting her own.
This trifling accident proves fatal. When a messenger
comes to take Mr. Soames* luggage to Dinard she
loseB the ferry-boat while seeing to her box. She
follows by the next boat, but he ha3 just gone to
Dinan, and when she tracks him there it is to find that he-
has started off for Mont St. Michael. There, at least, it
seems that she has overtaken him. He is not, indeed, at the
hotel, but he has merely gone for a walk through the town,
and will return to dinner. Comparatively at ease she lies
down to take a few hours' sleep, only to be awakened by the
noise of the departure of the capricious Soames. She followa
in all haste, but her carriage is overtaken by the tide ; she
and her driver are washed ashore, hut the horse is drowned,
and the next stage on the way to Dinan has to be made on,
foot.
Discouraged, at last she resolves to return to Southampton,
and on the voyage back befalls the moat humiliating of alt
her adventures. She makes the acquaintance of a young
Sandhurst cadet who proves to be a friend of her brother's.
She mentions that she is Graham Wendell's sister, but omita
to say that she is married. Therefore when, at Southampton,
the two are arrested as a dishonest clerk and an eloping wife
who have run away together, the fact that the young man-
gives her name as Miss Wendell and she herself as Mrs.
Leslie Somers, seem to afford sufficient ground for their being
consigned to prison, not without suffering from an insolence
of behaviour which we hope does not usually characterize
the British policeman.
Miss Somers if, however, communicated with, and brings
release and good tidings therewith. Lawrence Soames haa
been run to earth, and is now safe in Lord Wartlebury's
yacht. But the last disappointment iSf 'yet to come. When*
for the first time she comes face to face with him. Gertrude
finds that Lawrence Soames is not her husband.
Where, then, is Leslie Somers ? In the Trewport lodging,
though it would hardly be fair to say that he is safe. He had
hardly got out of sight of his wife when he fell down a hidden
ravine, and landed unconscious, with a broken ankle,
thirty feet below. There he was found by a deaf anri dumb-
shepherd, who kept him half a patient and half a prisoner
for several days. At last he escaped and made his way home*,
to find only his chfld there, his wife having gono to scour the
world in search of him. To Trewport, however, she return?
in despair, feeling that the boy is all that is left to her ; and
the scene closes with re-union and restored happiness.
It will be seen that the story has incident enough to please
even a school boy. It is, perhap3, improbable, but one does
not think of that until one has finished it. There is no deep-
character sketching, though one realizes Gertrude's tireless
devotion and " pluck," but the recital goes on with a-
constant vivacity that keepB up interest at the expense,
perhaps, of lessening emotion. Mrs, Grand can, aa we know,
attack serious problems in a serious spirit; it ia pleasant to
find that she has not thereby lost the power, as so many of
our noveliits seem to do, of telling a brisk and entertaining
romancs, every page of which it is a pleasure to read.
May 27 1893. THE HOSPITAL N URSING SUPPLEMENT. lxxxix
Zbe Book TKHorlb for Momen artfc Itturses.
tWe invite Correspondence, Oriticism, Enquiries, and Notes on Books likely to interest Women and Nurses. Addrers, Editor, The Hospital
(Nurses' Book World), 428, Strand, W.O.I
^Recollections of an Egyptian Princess. By Miss E.
Chennells (William Blackwccd and Sons).
Miss Chennells' record of her experiences during five
years' residence at the Court of the Khedive Ismael Pasha
is very pleasant reading. So often we are asked for Bome*
'thing to read aloud in the evening, not a novel. These
volumes furnish exactly the combination of amusement and
information which is commonly desired for this purpose.
?Few Europeans have had such opportunities as Mies Chennells
has enjoyed of studying the life of the harem from within,
and no one can fail to follow with interest her account of the
gradual development of her little Egyptian pupil, the Prin-
cess Zeynib, till her early marriage and sudden death. The
Khedive has done much to advance the cause of woman's
education by founding schools for girls, where a liberal
training is afforded. Perceiving that the education of his
own children could not be carried on satisfactorily in the
harem, beformcd an arrangement for them to spend thegreater
part of each day at a neighbouring house, where a complete
educational staff resided, and here Miss Chennells taught
ier little Princess until the time came when by Mahom-
midaa custom she was perforce "shut up" in the harem.
The lessons were then continued at the harem, though not
without many interruptions, among the most serious of
which may be reckoned the betrothal and marriage of the
fourteen year-old pupil. After the marriage Miss Chennell
yielded to the persuasion of the young bride, and took up
her residence in the harem in order to continue the course of
study already begun and lessen the tedium of the perpetual
seclusion. " I could not help being struck even at this early
stage, with the different life led by a young mariied couple
in the East and one in our country. My pupil's husband
had been in love with her from a child, and was devoted to
her after marriage as he had been before. Still, they had
no pursuits in common; they could not walk out together,
ride, drive, or go to the theatre together, or have any mutual
acquaintance. Any wish she might express was immediately
gratified by him; one pet after another was given
her; the poor child wanted liberty as a bird pines
in its cage, and cared for nothing else." The daily
routine of the harem, harmless, if childish, is related in
detail. The aimless hours of absolute idleness, the incessant
noiBy performances of the band, the total want of privacy
with slaves crowding round at every moment of the day, the
heat, the rigid seclusion from the world, and petty etiquette,
make up a picture unattractive enough. But in the midst
of all this it is encouraging to hear of the Princess persever-
ing in attempts at serious study, and taking keen delight in
Sir Walter Scott's novels, in " lies Miserables" and
Undine. "When a book was finished she spent a day or
two talking about the characters in it as if they had been
familiar friends, and was unwilling to begin another, as she
was sure she should never be so interested again." "Her
anxiety to do right was very marked. One day a visitor was
strongly recommending a book to her and offering to tend it
at once, when the FiincesB said, 'But is it pure?' The
visitor was rather out of countenance, and said to me, 'It
lc THE HOSPITAL NURSING SUPPLEMENT. Mat 27, 1893.
THE BOOS: WORLD FOR WOMEN AND NURSES-continued.
is by Dumas Fils !' " There seemed to be a brilliant future
of usefulness to her countrywomen, and pioneer work in
raiaiDg their position in the East, before her. But she had
always been delicate, and an attack of typhoid fever during
the first year of her married life put an end to the hopes
which centred round her.
After Twenty Years, anb Other Stories, By Julian
Sturgis (Longmans).
We gladly welcome this little series of short stories
collected from various magazines. There ia a charm which
it is not very easy to analyse about Mr. Sturgis's writing.
He dispenses almost entirely with plots, and his heroines are
merely indicated. But the middle-aged and scholarly
persons, about whom it is mostly his pleasure to write, ar3
excellent company, with this advantage moreover that they
are not met everywhere. Original, piquant, often disap-
pointed in life, and conscious of failure, Mr. Sturgis's
heroes have all that unconscious touch of the heroic
which is lacking to many more ambitious heroes, while the
humour is of the kind which is nearer akin to tears than
laughter. The little sketch entitled " The Philosopher's
Baby" is specially charming. The philosopher is being
congratulated on his approaohing marriage by a fellow
professor. " ' In the first place,' said he, ' I am sure that
instead of increasing my domestic worries my marriage will
transfer them in a body to my wife ; and, secondly, when I
consider the vast number of fools who are every day born into
the world, I am terrified by the picture of what the next genera-
tion will be if the thinkers of this are to be without
successors.' Having discharged his reasons in this wise, the
orator stood blinking at me, as if he feared dissent; but I was
too astounded by his magnificent audacity to reply. Slowly
a look of peace stole back into his face, a pleasant light
dawned in his eyes, and the promise of a smile at the corner
of his mouth. His remarkable fluency was gone, and,
indeed, his voice sounded quite choky when he said,
?Johnnie, you don't know what an angel she is.' A light
broke upon me. 'Philosopher,' I said, 'I believe you are
going to be married because you fell iD love.' ' Perhaps you
are right,' said the Philosopher." They go abroad for some
time after they are married, and the fellow philosopher hears
no news of them. Paying his first call after their return
home, he enters unannounced to the following scene : " My
friend's wife, the lady whom I had almost loved, queen
of all grace and comeliness, was appearing and dis-
appearing like a flash behind the day's Times, show-
ing at the moments of disclosure a faoe flashed with
excitement and lustrous coils of hair, tumbled into the
wildest disorder, while she accompanied the whole per-
formance with strange and inarticulate sounds. Her mother,
the same Mrs. Hanway, who was so perfect a model of
dresa and carriage that many of her lady friends were wont
to lament among themselves that she gave herself such airs,
was seated on the floor, dressed for walking, but without
her bonnet. Yes, she was certainly drumming on an in-
verted tea tray with the wrong end of the poker. And the
Philosopher ? It was perplexing after three years' separation
to meet him thus. The Philosopher was cantering round the
room on all fours, wearing on his head his own waste paper
basket. Briskly he cantered round, ever and anon frisking
like a lamb in springtime, until he reached my feet, which
were rooted to the ground in astonishment. Then in an
instant the cause of their eccentric conduct waB made clear.
Throned upon the hearthrug, and showing by a gracious
Bmile a few of the sweetest teeth, sat a fine baby of some
fifteen months. In one dimpled fist was tightly clinched the
brush which had so neatly arranged the mother's braids,
while the other was engaged in pounding the grandmother's
best bonnet into a shapeless maes." How the baby, other-
wise denominated " the noble woman," fell ill, and how the
Philosopher loBt his philosophy in the extremity of his grief,
and fell to praying like a common mortal is very beautifully
told. We have quoted enough to Bhow something of the
characteristics of Mr. Sturgis' methods to readers unac-
quainted with his previous worka.

				

## Figures and Tables

**Figure f1:**